# Comparison of Kompetitive Allele Specific PCR (KASP) and genotyping by sequencing (GBS) for quality control analysis in maize

**DOI:** 10.1186/s12864-015-2180-2

**Published:** 2015-11-06

**Authors:** Berhanu Tadesse Ertiro, Veronica Ogugo, Mosisa Worku, Biswanath Das, Michael Olsen, Maryke Labuschagne, Kassa Semagn

**Affiliations:** Ethiopian Institute of Agricultural Research (EIAR), Bako National Maize Research Center, Bako, West Shoa, Oromia Ethiopia; International Maize and Wheat Improvement Center (CIMMYT), P. O. Box 1041, Village Market, 00621 Nairobi, Kenya; Department of Plant Sciences, University of Free State, Bloemfontein, South Africa

**Keywords:** Genetic purity, Genetic identity, High marker density, Low marker density, Quality analysis, Single nucleotide polymorphism

## Abstract

**Background:**

Quality control (QC) analysis is an important component in maize breeding and seed systems. Genotyping by next-generation sequencing (GBS) is an emerging method of SNP genotyping, which is being increasingly adopted for discovery applications, but its suitability for QC analysis has not been explored. The objectives of our study were 1) to evaluate the level of genetic purity and identity among two to nine seed sources of 16 inbred lines using 191 Kompetitive Allele Specific PCR (KASP) and 257,268 GBS markers, and 2) compare the correlation between the KASP-based low and the GBS-based high marker density on QC analysis.

**Results:**

Genetic purity within each seed source varied from 49 to 100 % for KASP and from 74 to 100 % for GBS. All except one of the inbred lines obtained from CIMMYT showed 98 to 100 % homogeneity irrespective of the marker type. On the contrary, only 16 and 21 % of the samples obtained from EIAR and partners showed ≥95 % purity for KASP and GBS, respectively. The genetic distance among multiple sources of the same line designation varied from 0.000 to 0.295 for KASP and from 0.004 to 0.230 for GBS. Five lines from CIMMYT showed ≤ 0.05 distance among multiple sources of the same line designation; the remaining eleven inbred lines, including two from CIMMYT and nine from Ethiopia showed higher than expected genetic distances for two or more seed sources. The correlation between the 191 KASP and 257,268 GBS markers was 0.88 for purity and 0.93 for identity. A reduction in the number of GBS markers to 1,343 decreased the correlation coefficient only by 0.03.

**Conclusions:**

Our results clearly showed high discrepancy both in genetic purity and identity by the origin of the seed sources (institutions) irrespective of the type of genotyping platform and number of markers used for analyses. Although there were some numerical differences between KASP and GBS, the overall conclusions reached from both methods was basically similar, which clearly suggests that smaller subset of preselected and high quality markers are sufficient for QC analysis that can easily be done using low marker density genotyping platforms, such as KASP. Results from this study would be highly relevant for plant breeders and seed system specialists.

**Electronic supplementary material:**

The online version of this article (doi:10.1186/s12864-015-2180-2) contains supplementary material, which is available to authorized users.

## Background

In sub Saharan Africa (SSA), maize (*Zea mays* L.) is a staple food for more than 300 million people and is commonly grown by small-scale and resource-poor farmers in rural areas [1]. In Ethiopia, maize is the largest and most productive crop. In the 14 years period between 2000 and 2013, total annual production ranged from 2.7 to 6.7 million tons (http://faostat3.fao.org). During the same period, (i) maize yield in the country doubled from 1.6 t ha^−1^ in 2000 to 3.2 t ha^−1^ in 2013; and (ii) after eight years of erratic production, grain yield showed a rapid increase since 2007 [2]. However, productivity still remains far below the potential due to several factors, including periodic drought, high incidence of biotic stresses (diseases, insect-pests and parasitic weeds), poor soil fertility, scarcity of irrigation water, and inadequate farmer access to affordable quality seeds and fertilizers.

The formal state maize breeding program of Ethiopia was established in the early 1950’s and was instrumental in the development, evaluation and recommendation of adapted open pollinated varieties (OPVs). After nearly four decades, the breeding program released its first top cross hybrid, BH140, in 1988 [3] Subsequently, several high yielding and stress tolerant OPVs and hybrids adapted to different agro-ecologies have been released. These hybrid varieties, in conjunction with recent hybrids from private seed companies have significantly contributed to the current sharp increase in maize production in the country.

Initial adoption of hybrids by resource poor farmers was very slow for a number of reasons, including (i) high cost of hybrid seed relative to OPVs (especially as seed of OPVs can be recycled) (ii) limited or no access to improved hybrid seed in some regions; (iii) inadequate knowledge on agronomic management; (iv) insufficient seed companies and seed regulations in the country; (v) inadequate seed production infrastructure; and (vi) high cost of fertilizers [4]. The demand for hybrid seed gradually increased in Ethiopia as a result of changes in government policy including, but not limited to, the establishment of several local seed companies and the launching of a national extension program by government and non-governmental organizations (NGOs), such as Sasakawa Global 2000. The extension programs have made significant contribution in awareness creation of hybrid seed through field demonstration and providing technical support on hybrid maize grain production. Such rapid growth in hybrid adoption, however, brought a major concern on the quality of hybrid seed sold to resource poor farmers. Farmers reported high level of mixture of plants in their fields, and low yield in a given area. Despite the increased number of actors in the seed production and marketing venture, a vibrant national seed regulatory body to undertake effective seed quality assurance, including seed inspection and certification has been missing. Routine inspection of the initial parental seed (breeder, pre-basic and basic seed) produced by different actors in the seed value chain is critical and often done by inspecting production fields at vegetative and flowering stages. However, inspection of seed production fields based on a limited number of morphological and agronomic traits is time consuming, laborious, expensive, and at times can lead to inaccurate conclusions. Verification of seed lots and seed production fields can be effectively improved through the use of quality control (QC) genotyping using molecular markers.

Inbred lines are assumed to be genetically pure and possess all the genetic qualities that a breeder has selected for. Small changes in allele frequencies may occur during seed regeneration and maintenance breeding, and possible contamination with seeds or pollen of other samples [5, 6] Significant changes in the genetic makeup of a line may affect performance, and in the worst scenario result in distribution of wrong hybrids. Maintenance of inbred line genetic purity (homogeneity) and confirmation of the genetic identity of the same inbred line maintained at different locations are therefore important QC functions in maize breeding programs [7]. Several authors [5, 8–16] have reported the presence of a wide range of genetic differences among different seed sources of the same line designation. A high degree of differences among different seed sources of the same inbred line was also reported for some CIMMYT lines [7]. Thus breeding programs and seed distribution organizations must monitor the quality of seed increase and line maintenance processes using reliable tools to maintain the genetic homogeneity and identity of their key germplasm.

Single nucleotide polymorphic (SNP) markers have emerged as powerful tools for many genetic applications, including germplasm characterization (genetic diversity, genetic relationship, and population structure), QC analysis (genetic identity, genetic purity, and parentage verification), quantitative trait loci (QTL) mapping, allele mining, marker-assisted backcrossing, marker-assisted recurrent selection, and genomic selection. SNP data can be obtained using one of the numerous uniplex or multiplex SNP genotyping platforms that combine a variety of chemistries, detection methods, and reaction formats. Kompetitive Allele Specific PCR (KASP) is a uniplex SNP genotyping platform, and has developed into a global benchmark technology. CIMMYT uses a subset of 100 to 200 SNPs for routine QC genotyping using the KASP platform at LGC Genomics in the UK. The subset of SNPs for QC genotyping were selected out of the 1536 Illumina GoldenGate random chip using the following criteria: (1) ease of scoring with unambiguous separation of the two homozygous and heterozygous genotypes; (2) a minor allele frequency (MAF) and polymorphism information content (PIC) of at least 0.20 and 0.25, respectively; (3) good distribution across chromosomes based on physical position, and (4) good polymorphism across a wide range of mapping populations. The genotyping cost for KASP depends on the number of data points (1 data point = 1 sample genotyped by 1 SNP) and data turnaround, which is 4 to 6 weeks for normal turnaround and 2 to 3 weeks for rapid turnaround. The current KASP genotyping cost for normal and rapid data turnaround ranges from US$0.064 to US$0.242 and from US$0.100 to US$0.360, respectively [7]. Genotyping by sequencing (GBS) [17] is an alternative method that could be used in generating high density genotype data at a genotyping and allele calling cost ranging from $18 to $38 per sample, depending on the level of multiplex (http://www.biotech.cornell.edu/brc/genomic-diversity-facility/services/gbs-project-design-and-optimization). To our knowledge, however, the correlation between GBS and KASP markers for QC analyses is not known. The objectives of our study were to 1) evaluate the level of genetic purity within each inbred line and understand genetic identity among different seed sources of the same line designation; and 2) compare the correlation between the KASP-based low density and the GBS-based high density information for QC analysis.

## Methods

### Sample preparation and genotyping

A total of 80 samples from 16 inbred lines, which are parental lines of eight popular Ethiopian hybrids (BH140, BH540, BHQP542, BH543, BHQPY545, BH660, BH670, and BH661), were used in this study (Table 1). Each inbred line was represented by from two to nine seed sources collected from the maize breeding program of the Ethiopia Institute of Agricultural Research (EIAR), seed companies in Ethiopia, the Ethiopian Institute of Biodiversity Conservation (IBC) (the national gene bank), and the International Maize and Wheat Improvement Center (CIMMYT) (Table 1). The seed samples from IBC were used as reference for older EIAR inbred lines, while the seeds obtained from CIMMYT were used to compare with the corresponding line designation maintained by EIAR and partners in Ethiopia. Seedlings were raised on plastic trays at the Biosciences eastern and central Africa (BecA) hub screen-house in Nairobi, Kenya. A single leaf from each of ten plants per sample were piled together, the tips trimmed off and approximately equal amount of leaf segment cut at once to make a bulk, and transferred into 1.2 mL strip tubes that contained two 4-mm stainless steel grinding balls (Spex CetriPrep, USA). Genomic DNA was extracted using a modified version of the CIMMYT high throughput mini-prep Cetyl Trimethyl Ammonium Bromide (CTAB) method as described elsewhere [18]. This extraction protocol has longer steps but provides good quality DNA for different purposes, including GBS that involves restriction digestion. DNA concentration was measured using the Quant-iT™ PicoGreen® dsDNA assay kit (Invitrogen™, Paisley, UK) and the Tecan Infinite F200 Pro Plate Reader (Grödig, Austria), and normalized to 50 ng/μL. For GBS, the quality of the extracted DNA was checked by digesting 250 ng of the genomic DNA from 8 randomly selected samples with 3.6 units of ApeKI restriction enzyme (New England Biolabs, Boston, USA) at 75 °C for three hours. DNA samples were shipped to both LGC Genomics (http://www.lgcgroup.com) and the Genomic Diversity facility at Cornell University (http://www.biotech.cornell.edu/brc/genomic-diversity-facility). Samples were genotyped with 200 SNPs (Additional file 1) prioritized by CIMMYT for QC genotyping using KASP genotyping platform [7]. The same DNA samples were also genotyped using GBS as described by Elshire and colleagues [17]. GBS data was generated by the Genomic Diversity Facility, Cornell University using *ApeKI* as restriction enzyme and 96-plex multiplexing.

**Table 1:** Proportion of homogeneity (purity) for 80 samples from 16 inbred lines using 191 KASP and 257,268 GBS markers

No	Name	Seed class	Source	Homogeneity (%)	
				KASP	GBS
1	SC22_AR	Basic	AgriCEFT	65	79
2	SC22_AR09	Pre-basic	AARC	59	76
3	SC22_BARC2011k	Basic	BARC	66	80
4	SC22_BARC2012k	Basic	BARC	69	81
5	SC22_EB	Nucleolus	BNMR	79	86
6	SC22_GB	Gene bank	Gene Bank	73	82
7	SC22_HD04/05	Pre-basic	HARC	63	79
8	SC22_I11B	Pre-basic	BNMR	67	81
9	SC22_I12B	Pre-basic	BNMR	64	80
10	124-b(113)_AR09	Pre-basic	AARC	88	88
11	124-b(113)_ASE	Basic	ASE	76	83
12	124-b(113)_BARC2010k	Basic	BARC	82	85
13	124-b(113)_BARC2011k	Basic	BARC	84	85
14	124-b(113)_EB	Nucleolus	BNMR	88	89
15	124-b(113)_GB	Gene bank	Gene Bank	89	89
16	124-b(113)_HD	Pre-basic	HARC	75	82
17	124-b(113)_HD04/05	Pre-basic	HARC	69	80
18	124-b(113)_I12K	pre-basic	BNMR	83	85
19	124-b(109)_EB	Nucleolus	BNMR	68	81
20	124-b(109)_HD04/05	Pre-basic	HARC	58	74
21	124-b(109)_I11B	Pre-basic	BNMR	59	74
22	124-b(109)_I13B	Pre-basic	BNMR	56	74
23	CML197	CIMMYT	CIMMYT	91	100
24	CML197_EB	Nucleolus	BNMR	73	85
25	CML197_HD03/04	Pre-basic	HARC	69	84
26	CML197_I10K	Pre-basic	BNMR	69	84
27	CML197_I11K	Pre-basic	BNMR	72	85
28	CML197_I12K	Pre-basic	BNMR	67	85
29	CML312	CIMMYT	CIMMYT	100	100
30	CML312_EB	Nucleolus	BNMR	99	100
31	A7033_AR02/03E.C	Pre-basic	AgriCEFT	61	79
32	A7033_AR08	Pre-basic	AARC	63	80
33	A7033_EB	Nucleolus	BNMR	75	85
34	A7033_GB	Gene bank	Gene Bank	76	84
35	A7033_HD	Pre-basic	HARC	61	82
36	A7033_I10K	Pre-basic	BNMR	62	80
37	A7033_I11K	Pre-basic	BNMR	65	81
38	A7033_selected	Breeder seed	BNMR	70	84
39	F7215_AR02/03E.C	Pre-basic	AgriCEFT	63	77
40	F7215_AR08	Pre-basic	AARC	69	78
41	F7215_EB	Nucleolus	BNMR	77	82
42	F7215_GB	Gene bank	Gene Bank	64	78
43	F7215_HD04/05	Pre-basic	HARC	67	77
44	F7215_I11K	Pre-basic	BNMR	65	77
45	F7215_I12K	Pre-basic	BNMR	67	78
46	F7215_selected	breeder seed	BNMR	70	80
47	142-1-e_AR02/03E.C	Basic	AgriCEFT	82	83
48	142-1-e_EB	Nucleolus	BNMR	89	86
49	142-1-e_GB	Gene bank	Gene Bank	89	87
50	142-1-e_I10K	Pre-basic	BNMR	85	85
51	142-1-e_I12K	Pre-basic	BNMR	87	84
52	CML202	CIMMYT	CIMMYT	99	100
53	CML202_12K	Pre-basic	BNMR	99	99
54	CML202_EB	Nucleolus	BNMR	99	99
55	CML202_HD04/05	Pre-basic	HARC	96	99
56	CML202_I11K	pre-basic	BNMR	92	98
57	CML395	CIMMYT	CIMMYT	100	100
58	CML395_12K	Pre-basic	BNMR	64	81
59	CML395_EB	Nucleolus	BNMR	49	77
60	CML395_HD	Pre-basic	HARC	60	81
61	CML395_I11K	Pre-basic	BNMR	59	81
62	144-7-b_I08K	Pre-basic	BNMR	80	84
63	144-7-b_I09k	Pre-basic	BNMR	79	83
64	144-7-b_I10k	Pre-basic	BNMR	81	84
65	CML144	CIMMYT	CIMMYT	99	100
66	CML144_EB	Nucleus	BNMR	98	99
67	CML144_I11B	Pre-basic	BNMR	98	98
68	CML144_I13B	Pre-basic	BNMR	96	97
69	CML159_EB	Nucleolus	BNMR	98	99
70	CML159_I09K	Pre-basic	BNMR	93	97
71	CML159_I13B	Pre-basic	BNMR	90	96
72	CML176_EB	Nucleolus	BNMR	87	94
73	CML176_I12K	Pre-basic	BNMR	83	94
74	CML161	CIMMYT	CIMMYT	100	100
75	CML161_I12B	Pre-basic	BNMR	99	100
76	CML161_I13B	Pre-basic	BNMR	100	100
77	CML165	CIMMYT	CIMMYT	98	100
78	CML165_EB	Nucleolus	BNMR	95	98
79	CML165_I11B	Pre-basic	BNMR	98	99
80	CML165_I12B	Pre-basic	BNMR	88	93

### Data analyses

The raw allele calls received from LGC Genomics consisted of several unassigned SNP calls. To minimize the unassigned calls, the KASP data was rescored using Kluster caller software from LGC Genomics. Nine SNPs were excluded either due to large missing data points or ambiguity in clearly discriminating the homozygous and heterogeneous genotypes so analyses were conducted using 191 of the 200 SNPs. Since GBS generates a high percentage of un-called genotypes, the missing data was imputed by the Institute of Genomic Diversity (IGD), Cornell University using an algorithm that searches for the closest neighbor in small SNP windows across the maize database [19]. Imputed data for 955,120 loci was received, but the majority of the GBS markers were monomorphic. The imputed GBS data was filtered using a minor allele frequency (MAF) of 0.05 in TASSEL version 4.3.2 software [20], yielding 257,268 polymorphic SNPs (26.9 % of the initial loci) for further analyses (Table 2). The proportion of missing data after filtering with a MAF of 0.05 varied between 2.6 and 14.4, and the overall average across the 80 samples was 7.3 %.

**Table 2:** Summary of the number of KASP and GBS markers used in the present study

Chromosome	GBS	KASP
1	40,666	26
2	31,600	21
3	30,120	19
4	23,977	19
5	29,656	19
6	20,880	17
7	21,084	16
8	21,651	19
9	19,886	19
10	17,748	16
Total	257,268	191

Eight datasets were used for all statistical analyses. Dataset 1 and dataset 2 consisted of the 191 KASP SNPs and the 257,268 GBS markers after filtering using a MAF of 0.05, respectively. Six additional input files were later created for correlation analyses: (a) dataset 3 consisted of a subset of 100 out of 191 SNPs routinely used by CIMMYT for QC genotyping; and (b) data set 4 to dataset 8 were created from dataset 2 using a MAF of 0.10, 0.20, 0.30, 0.40 and 0.50. For QC analysis, the best SNPs would be those which amplify the two alleles equally (i.e., each with a frequency of 0.50). The proportion of heterogeneity (the number of markers that were not homozygous due to mixture of two homozygous genotypes or residual heterozygosity) in each sample was calculated from all datasets using TASSEL version 4.3.2. Genetic purity was calculated from all datasets in Excel as the difference between 100-h, where h refers to heterogeneity in percentage obtained from TASSEL. For all eight datasets, genetic distance was calculated between each pair of samples using the identity by descent (IBS) method implemented in TASSEL. Dendrograms were constructed from the genetic distance matrices of both dataset 1 and dataset 2 using the Unweighted Pair Group Method with Arithmetic Mean (UPGMA) algorithm implemented in molecular evolutionary genetics analysis (MEGA), version 6 [21]. Mantel tests [22] were used to compute the correlation between the genetic distance matrices derived from all eight datasets using NTSYS-pc (numerical taxonomy and multivariate analysis system), version 2.11 [23]. Pearson correlation coefficients between genetic purity values obtained for all eight datasets were calculated using MINITAB v14.

## Results and discussion

### Genetic purity (homogeneity)

**Fig. 1:**
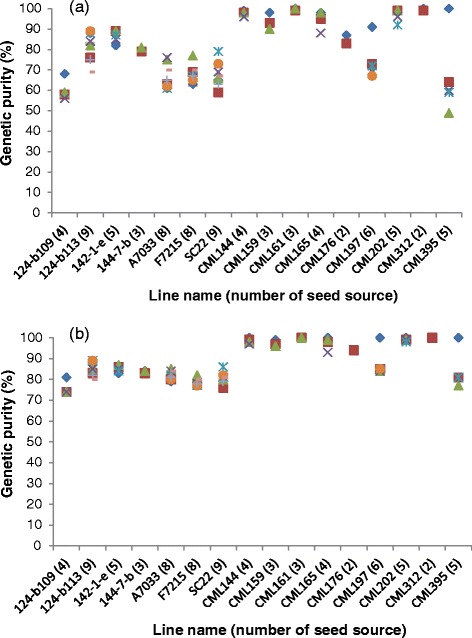
Comparison of genetic purity of multiple seed sources of the 16 inbred lines using **a** 191 KASP and **b** 257,268 GBS. For each line designation, the number of seed sources is shown in the x-axis in bracket and with different shapes in the plot

**Fig. 2:**
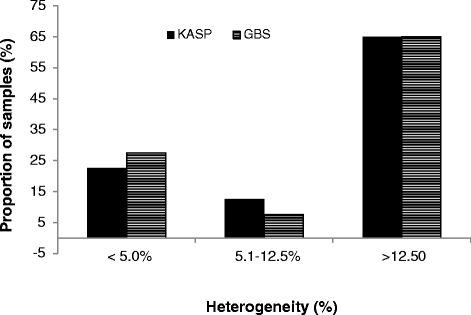
Summary of the heterogeneity of 80 seed sources from 16 inbred lines based on 191 KASP and 257,268 GBS markers. See Table 1 for homogeneity values of each marker

We first computed the proportion of homozygous SNPs on each of the 80 samples as an estimate of genetic purity or homogeneity. The results were highly variable across samples, with homogeneity varying from 49 to 100 % for KASP and from 74 to 100 % for GBS (Table 1; Fig. 1). The overall average homogeneity across all 80 samples was 79 % for KASP and 87 % for GBS. Most breeding programs now use inbred lines at F_4_ or later generations, but previously, lines were often derived at earlier generations. An inbred line may be considered pure or homogenous if the proportion of heterozygous or heterogeneous loci does not exceed 5 % [7]. Samples with substantially more than 5 % heterogeneity (3.1 % due to residual heterozygosity in the founder plants plus 1.9 % due to both genotyping error and genetic drift) for a given set of SNPs are likely to have been contaminated by pollen or seed of another genotype. In the present study, approximately 23 % of the samples in KASP and 28 % of the samples in GBS were considered genetically pure with ≤5 % heterogeneity (Fig. 2). The majority of the samples (77 % in KASP and 72 % in GBS) showed high proportion of heterogeneity that varied from 6 to 51 % in KASP and from 6 to 26 % in GBS.

**Fig. 3:**
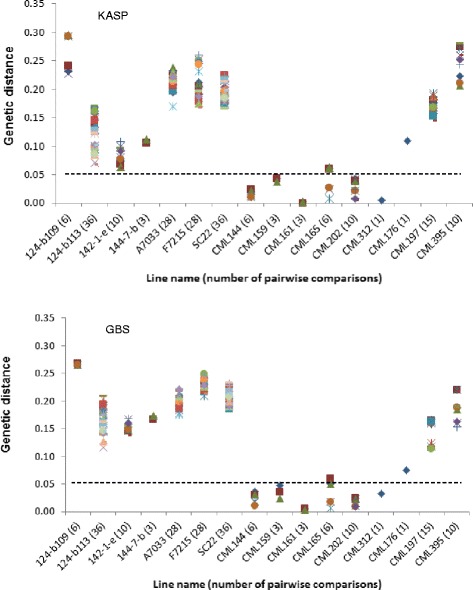
Summary of pairwise comparisons of genetic distance among multiple sources of the same line designation genotyped with 191 KASP and 257,268 GBS markers. For each line designation, the number of seed sources is shown in the x-axis in bracket and with different shapes in the plot

Genetic purity among the multiple seed sources of each of the 16 inbred lines was compared to understand whether the high proportion of heterogeneity was specific to a few lines or common across most lines (Fig. 3). Genetic purity was consistently lower for all inbred lines developed by EIAR irrespective of their seed origin or marker density. The genetic purity of CIMMYT lines maintained at EIAR and partners was highly variable, with some showing much lower than the expected level of purity, while others had high level of purity. For example, the seed sources obtained from EIAR and partners for both CML395 and CML197 showed the lowest purity (49 and 73 % for KASP and 77 and 85 % for GBS, respectively), while CML312, CML202, CML144 and CML161 from EIAR and partners showed 96 to 100 % purity regardless of sources and marker density (Table 1). All CML seed sources obtained from CIMMYT showed the highest purity, which varied between 98 and 100 % for both KASP and GBS except CML197 that showed conflicting results for KASP (91 %) and GBS (100 %).

Results above highlight two main points. First, the level of purity for most samples originating from EIAR and partners was low because most inbred lines sampled from these sources were early generation inbred lines used as parents for old commercial hybrids in Ethiopia. Prior to the release of hybrids, maize farmers in the country used to grow OPVs for several reasons, including the relatively higher cost of imported hybrid seeds and higher input recommendation for hybrids as compared to OPVs [24]. In addition, the source germplasm which was available for line development at the time was unimproved and intolerant to inbreeding depression. To cope with these challenges, breeders of the time opted to develop and release hybrids using early generation parental inbred lines [3]. Although this strategy favors cheaper seed production and hybrids derived from such early generation parental lines out-yielded OPVs, generally they remain inferior in uniformity as compared to hybrids developed from fixed lines.

Second, the level of purity from the present study clearly agrees with the stage of inbreeding and our field observation on lack of uniformity for most of the older EIAR inbred lines. Complaints from growers on the unexpectedly high level of variability under farmers’ field conditions is associated with a combination of genetic reasons, handling of early generation inbred lines and inadequate seed inspection and quality assurance system for hybrid seed production in the country. However, mislabeling, pollen contamination and seed admixture are some of the other major reasons that might have contributed for the variation observed under the farmers’ field.

### Genetic identity

**Fig. 4:**
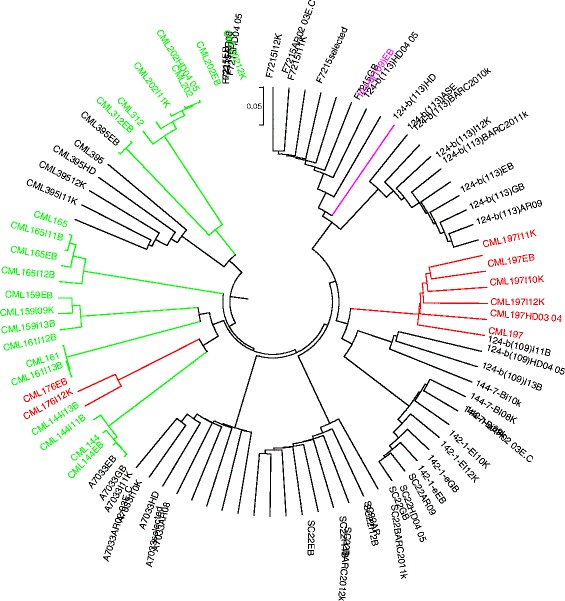
UPGMA dendrogram of 80 samples from 16 inbred lines based on genetic distance matrix computed from 191 KASP SNPs

**Fig. 5:**
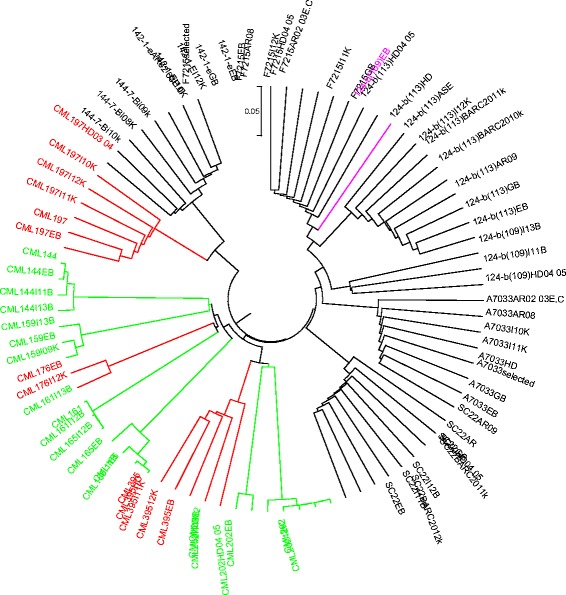
UPGMA dendrogram for 80 seed sources from 16 inbred lines based on genetic distance matrix computed from 257,268 GBS markers

Multiple seed sources of a given inbred line developed after four generations of inbreeding are expected to be genetically identical or nearly so with a maximum allelic difference of <5 % [7]. In the present study, genetic distance among the different seed sources of the same line designation varied from 0.000 to 0.295 for KASP and from 0.004 to 0.230 for GBS. As shown in Fig. 3, the genetic distance among all pairewise comparisons of seed sources for five CMLs (CML144, CML159, CML161, CML202 and CML312) was < 0.05. The remaining eleven inbred lines, including four CMLs and all seven lines from EIAR, showed higher proportion of mismatch for two or more seed sources. The dendrograms in both Figs. 4 and 5 also clearly show the extent of genetic differences among multiple seed sources of the same line designation. Importantly, dendrograms from both KASP and GBS datasets have grouped different seed sources of the same line designation together except 124-b (109) EB. The grouping of all sources of the same line designation together is an indication that all sources of the same line were derived from the same origin, but most of them have diverged significantly for several reasons, including residual heterozygosity, seed or pollen contamination, genetic drift and the method of line maintenance. Labeling error is the most likely explanation for one of the samples of 124-b (109) EB mis-grouping from the other sources (Figs. 4 and 5). The effect of method and frequency of maintenance on genetic structure of heterozygous individuals is hastened by natural and artificial selection, which entails the elimination of individuals carrying undesirable alleles [5, 11, 13]. To maintain allele frequencies in early generation inbred lines, it is compulsory to raise large population sizes in the sibbing blocks in order to avoid genetic drift. In standard line maintenance, however, the breeders would most likely be using few plants which can affect the identity of early generation lines amongst sources due to drift and selection bias. Because of this practical difficulty, EIAR breeders have been incrementing pre-basic/basic seed of all early generation inbred lines only in isolated blocks in order to minimize genetic drift. This method allows for frequent inspection of seed production fields along with rigorous rouging when off-type plants are observed; and has helped to maintain early generation lines for more than two decades enabling farmers to access true-to-type hybrid seed every season. Reliable seed production has contributed to the sharp increase in maize productivity in the country for food security. However, when seed companies started to produce their own pre-basic/basic seed in different locations, the supply of consistent hybrid seed to farmers become a challenge. Significant variation of performance of various seed lots of the same hybrid designation was observed, likely due to differences in parental line maintenance methods.

### Correlation between low and high marker density

**Fig. 6:**
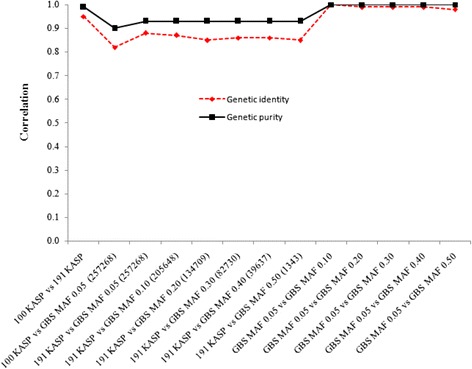
Correlation coefficients between different number of KASP and GBS markers for genetic purity and identity estimated from 80 samples

Semagn et al. [7] prioritized a subset of about 100 to 200 SNPs for routine QC analysis using KASP genotyping platform. In order to understand the relationship between marker types and densities using KASP or GBS markers for estimating genetic purity and identity, we conducted correlation analyses by creating several subsets of data (Fig. 6). The correlation between the subset of 100 and 191 KASP SNPs recommended for QC analysis by Semagn et al. [7] was 0.95 for identity and 0.99 for purity. When the 100 KASP markers were compared with the entire 257,268 GBS markers, the correlation coefficients were 0.82 for identity and 0.90 for purity. The increase in KASP markers from 100 to 191 increased the correlation with GBS by only 0.03 for purity and 0.06 for identity. The KASP and GBS markers showed some discrepancy in terms of numerical values when heterogeneity exceeded 12.5 %, with 61.3 % of the samples showing 12.5 to 25 % heterogeneity in GBS and 43.8 % of the samples showing >25 % heterogeneity in KASP (Fig. 3). However, the conclusions reached in assigning lines into genetically pure or not were highly similar. Given the low accuracy of GBS in correctly calling highly heterogeneous and heterozygous germplasm and some of the issues associated in imputing GBS calls, such discrepancies may be expected. A reduction in GBS markers from 257,268 to 1,343 did not impact correlation values for the various pairwise comparisons (Fig. 6). Therefore, the effect of marker density for routine QC analysis seems relatively minor. It is concluded that 100 to 200 SNP markers routinely used by CIMMYT for QC genotyping are sufficient for genetic purity and identity purposes. The use of high density GBS markers for QC analysis, at least at present, does not add value to the process for different reasons, including longer data turnaround time and lower accuracy in correctly calling alleles in highly heterogeneous and heterozygous germplasm.

### Implication of the QC results and recommendations

Overall, most seed sources from CIMMYT were considered genetically pure, which was not the case for the majority of samples originating from EIAR. Such results, however, are expected for inbred lines developed with only a few generations of inbreeding. One of the major objectives of maize breeders in Ethiopia during the 1980s was to develop maize germplasm that performed better than OPVs in order to address the outcry over food insecurity in the country. During that time, breeders had limited access to diverse maize germplasm for new pedigree starts, there were no private companies involved in maize breeding and/or seed multiplication and marketing in the country, and there were no clear seed regulations governing quality control and assurance. Breeders were pressured to release improved hybrids which could outperform widely grown OPVs. They used early generation inbred lines to develop and release hybrids, and maintained these lines in isolation increases without losing their identity. This contributed to sharp growth of maize production in the country. Currently, Ethiopia has a relatively well established seed law for QC/QA using morpho-agronomic traits. This can be strengthened by incorporating modern molecular tools. Maize breeders in the country also have better access to a wide range of germplasm for new pedigree starts from different national programs in Africa, CIMMYT, and the International Institute for Tropical Agriculture (IITA). They are now developing inbred lines after four or later generations of selfing and more recently released hybrids developed using inbred lines with higher genetic purity. This will assist to maintain homogeneous parental inbred lines in the next generation hybrids in Ethiopia.

The use of parental inbred lines with high heterogeneity in breeding programs can have multifold negative effects, including the use of wrong donors in new breeding starts for line development, hybrid formation as well as genetic and molecular studies. Genetic purity of a given parental line also has major impact on the production and distribution of certified seed to farmers. Failure to undertake regular quality control analysis could result in generation and dissemination of incorrect products to the end user. Maize breeders commonly exchange seed of the most widely used inbred lines; therefore, unexpected level of genetic heterogeneity in a given seed lot or high mismatch across different seed sources of a given inbred line can quickly spread across different programs with consequent negative effects. Obtaining seed from a reliable source and undertaking routine quality control, will be useful for minimizing errors associated with purity and identity. Our results showed that a subset of 100 to 200 SNP (KASP) markers would be sufficient for routine QC analysis. In cases where there is no reference genotype data for inbred lines that will be used for comparison purposes, it is recommended to grow out multiple sources of different sources of the same line designation in nurseries, generate SNP data, compare SNP data with field notes, and discard those sources that show deviation from expectation in terms of purity and identity. CIMMYT has carried out this exercise to resolve genetic purity and identity issues from 280 seed sources involving 40 inbred lines and strongly recommends this regular activity for other breeders (M. Worku, personal communication). The information presented in this paper would be highly useful for maize breeders that are involved in new pedigree start, developing populations for QTL mapping and marker-assisted breeding, and the seed companies.

## Conclusions

Our results clearly showed high discrepancy both in genetic purity and identity by the origin of the seed sources (institutions) irrespective of the type of genotyping platform and number of markers used for analyses. Overall, most seed sources from CIMMYT were considered genetically pure, which was not the case for the majority of samples originated from EIAR. One of the reasons for such discrepancy in genetic purity and identity was the level of inbreeding prior to releasing the parental lines for hybrid formation. Although there were some differences between KASP and GBS results, the overall conclusions reached from both methods was basically similar, which clearly suggests that smaller subset of preselected high quality markers are sufficient for QC analysis that can easily be done using low marker density genotyping platforms, such as KASP. GBS data would be highly useful for establishing reference marker database at the time of releasing an inbred parental line for used in heterotic grouping and planning hybrid combinations and new pedigree starts. Results from this study would be highly relevant for plant breeders and seed system specialists.

## Additional file

Additional file 1:
**Summary of the 200 KASP SNPs used for genotyping.** (XLSX 17 kb)

## Abbreviations

CIMMYT: International maize and wheat improvement center
CTAB: Cetyl trimethyl ammonium bromide
EIAR: Ethiopian Institute of Agricultural Research
GBS: Genotyping by sequencing IBS, identity by descent
IGD: Institute of genomic diversity
KASP: Kompetitive Allele Specific PCR MAF, minor allele frequency
MEGA: Molecular evolutionary genetics analysis
NGOs: Non-governmental organizations
OPVs: Open-pollinated varieties
QC: Quality control analysis
SNP: Single nucleotide polymorphism
SSA: Sub Saharan Africa
UPGMA: Unweighted pair group method with arithmetic mean
